# Genomics and Chlamydial Persistence *In Vivo*

**DOI:** 10.1128/mBio.02616-19

**Published:** 2019-12-17

**Authors:** Dan D. Rockey, Robert J. Suchland, Steven J. Carrell

**Affiliations:** a Oregon State University, Corvallis, Oregon, USA; b University of Washington, Seattle, Washington, USA; Sequella, Inc.

**Keywords:** chlamydia, persistence, *trpA*

## LETTER

We read with interest the recent paper by Somboonna and colleagues, addressing changes to tryptophan synthase in a single Chlamydia trachomatis strain that had an aberrant growth phenotype *in vitro* (1). This strain was isolated four times, over 4 years, from a patient who was apparently persistently infected. This strain was highly related to a serovar F strain isolated previously in their clinical setting. The authors use these strains to defend an association between *in vivo* persistence and a particular mutation at the 3′ end of the *trpA* gene. We have two issues in this work that we address below.

First, the paper did not reference our recent report that characterized persistent C. trachomatis strains in five individual patients at Seattle/King County sexual health clinics that had persisted for up to 5 years (2). Their description of the serial isolation of a genomically identical serovar F strain four times from an individual patient is consistent with our report and adds strength to the concept that individual patients can be colonized by C. trachomatis even in the face of aggressive diagnostic efforts and antibiotic therapy. This is an important concept in sexual health research: the role of persistent chlamydial infections in patients has been modestly controversial and remains an active area of research interest. To this end, we are pleased to see other laboratories using a genome sequencing approach similar to ours, supporting the concept of *in vivo* persistence in female patients.

**FIG 1:**
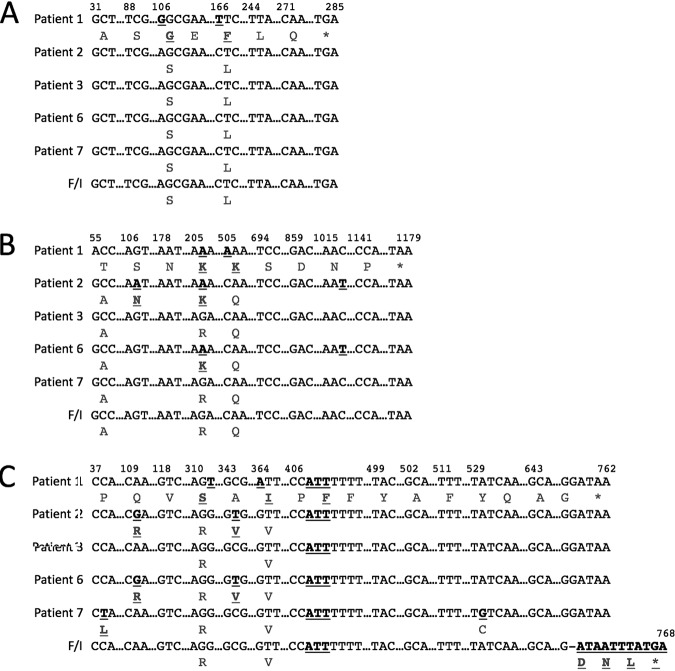
Mutations in *trpR* (A), *trpB* (B), and *trpA* (C) in five persistent strains from patients in sexual health clinics in King County, Washington (patients 1, 2, 3, 6, and 7) discussed in reference 2 and the F/I strain discussed in reference 1. The figure is formatted similarly to data in reference 1. Positions in each coding sequence are indicated at the top of each panel (the numbers are centered on the base), and amino acid sequence and changes are indicated below each DNA sequence. Mutant bases and amino acids are indicated by boldface type as in reference 1.

More importantly, we are concerned that the paper stresses a causative association between their identified mutation in *trpA* and the persistence phenotype in patients. We examined our five strains from persistently infected individuals and found that none of them had the mutation discussed in their paper (Fig. 1). We expanded that analysis to demonstrate that there were no unique mutations in the *trp* operon in any of our persistent strains, and no genetic evidence in all of the collected read sets that any of these strains were accumulating a minority population of mutations in this operon (not shown).

While the *in vitro* work described by these authors is interesting and convincingly discusses the tryptophan-related biology of their strain, we think it is important to be wary of the proposed causal effect between this *in vitro* property and persistence of C. trachomatis in patients.
